# Aggressive behaviour is affected by demographic, environmental and behavioural factors in purebred dogs

**DOI:** 10.1038/s41598-021-88793-5

**Published:** 2021-05-03

**Authors:** Salla Mikkola, Milla Salonen, Jenni Puurunen, Emma Hakanen, Sini Sulkama, César Araujo, Hannes Lohi

**Affiliations:** 1 Department of Medical and Clinical Genetics, University of Helsinki, Helsinki, Finland; 2 Department of Veterinary Biosciences, University of Helsinki, Helsinki, Finland; 3 Folkhälsan Research Center, Helsinki, Finland

**Keywords:** Behavioural ecology, Ecological epidemiology, Risk factors, Animal behaviour, Animal physiology

## Abstract

Aggressive behaviour is an unwanted and serious problem in pet dogs, negatively influencing canine welfare, management and public acceptance. We aimed to identify demographic and environmental factors associated with aggressive behaviour toward people in Finnish purebred pet dogs. We collected behavioural data from 13,715 dogs with an owner-completed online questionnaire. Here we used a dataset of 9270 dogs which included 1791 dogs with frequent aggressive behaviour toward people and 7479 dogs without aggressive behaviour toward people. We studied the effect of several explanatory variables on aggressive behaviour with multiple logistic regression. Several factors increased the probability of aggressive behaviour toward people: older age, being male, fearfulness, small body size, lack of conspecific company, and being the owner’s first dog. The probability of aggressive behaviour also differed between breeds. These results replicate previous studies and suggest that improvements in the owner education and breeding practices could alleviate aggressive behaviour toward people while genetic studies could reveal associated hereditary factors.

## Introduction

Aggressive behaviour is a serious and common behaviour problem in domestic dogs^1^. Aggressively behaving dogs can cause public concern by biting people and other pets, with medical or even lethal consequences for the victim. In some countries, certain dog breeds are even banned or are under breed-specific restriction in order to minimize the potential risk of dog bites^2,3^. Additionally, aggressive behaviour often leads to surrender or even euthanasia of the dog^4^, disposing the aggressively behaving individuals to welfare problems. Aggressive behaviour can also arise from pain^5,6^, suggesting that some aggressively behaving dogs may have a disease, such as hip dysplasia^7^, or other painful condition which impair their welfare.

The severity of aggressive behaviour varies from biting and snapping attacks that can even lead to the death of a victim to less severe, but more common growling and barking^8^. When including these less severe signs of aggressive behaviour, the aggressive behaviour toward people are quite common in pet dogs even though the reported proportions differ depending on the study approaches and study populations. In Iran, 26% of dogs showed aggressive behaviour toward strangers in a pilot study^9^, and in an English dog population 3% of dogs showed aggressive behaviour toward family member, and 5–7% toward strangers^10^. In a Finnish dog population, in the study of Tiira et al.^11^, the proportion of aggressive behaviour toward the owner/family members and toward strangers/familiar people were 16% and 45%, respectively. However, in our more recent prevalence study from Finnish dogs, aggressive behaviour was less common: the prevalence of total aggressive behaviour in this study population was 14%, aggressive behaviour toward (human) family members 6.4%, and toward strange people 6%^12^. The different criteria to categorise a dog as aggressive or non-aggressive explains the differences in the reported percentages of dogs showing aggressive behaviour. For example, in our more recent study^12^, we only considered dogs that had growled at least often or had tried to bite or snap at least sometimes as aggressive, while Tiira et al.^11^, considered all dogs that had barked, growled, snapped, or bit at least once as aggressive. Thus, in our study aggressive behaviour toward people includes frequent growling, snapping and biting or trying to snap or bite.

Aggressive behaviour in dogs has been associated with several factors. Some of these identified factors are dog-related, for example, dog’s fearfulness^11,13^, older age^10,14,15^, and being male^1,14^. The association with sterilisation is inconsistent, as studies have showed a lower probability of overall aggressive behaviour in sterilised than intact dogs^1^, a higher probability (toward owner) of aggressive behaviour in sterilised dogs^14^, and no connection between sterilisation and aggressive behaviour^14–16^. Some previous studies have also identified size as an affecting factor, with small dogs behaving more likely aggressively than large dogs^17,18^. Differences in aggressive behaviour between breeds have also been studied before, and several studies have detected significant breed-wise differences^11,12,14,19^. In addition, various environmental factors have been associated with aggressive behaviour. For example, dogs living in a single-dog household have been found to more likely behave aggressively toward the owner than dogs living in multi-dog household^14,20^, and dogs living in larger families have been found to be more prone to aggressive behaviour^14,15^. Dogs living in rural areas have been found to more likely behave aggressively toward strangers than dogs living in cities^14^. Furthermore, time spent with the owner^14^, and owner’s dog experience^14,20–22^ have been associated with aggressiveness, and early weaning has been suggested to increase the probability of aggressive behaviour^23^.

We studied the factors associated with canine aggressive behaviour toward people (strangers and family members) in over 9000 Finnish purebred pet dogs with multiple logistic regression and we also formed a priori hypotheses based on previous literature. The dataset we used in this study is part of our larger owner-completed online questionnaire data with over 13,700 dogs^12^. Reliability of questionnaires is usually good, reflecting the behaviour of a dog in behaviour tests^24,25^ and over time^25^. An owner-questionnaire can even be a better method to study aggressive behaviour than behaviour tests, because all dogs that have behaved aggressively in daily life do not show aggressive behaviour in test situations^26,27^. Here, our aim was to study the association of known (living environment, family size, dogs in the family, owner’s dog experience, daily exercise) and novel (daily time spent alone, weaning age) factors with aggressive behaviour in a previously unstudied dog population.

## Results

### Study cohort and demographics

We studied factors associated with aggressive behaviour in Finnish pet dogs with an owner-completed online questionnaire and collected a cross-sectional convenience sample of 9270 dogs, including 1791 dogs in the high and 7479 dogs in the low aggressive behaviour groups. The mean age of the dogs was 4.6 years (ranging from 2 months to 17 years) and 53% of them were female. The number of dogs in different breed, sex, and aggressive behaviour groups are shown in the Supplementary Table S1. We have a manuscript about study participants in preparation.

### Factors associated with aggressive behaviour

The final logistic regression model for aggressive behaviour included explanatory variables age, sex, fearfulness, breed, dogs in the family, body size, and owner’s dog experience (Table 1).

**Table 1:** Association between the explanatory variables and aggressive behaviour in the logistic regression model.

Explanatory variable	DF	χ^2^	*P*-value
Intercept	1	89.547	**< 0.0001**
Age	1	4.575	**0.0324**
Sex	1	90.498	**< 0.0001**
Fearfulness	2	596.059	**< 0.0001**
Breed	22	102.448	**< 0.0001**
Dogs in the family	1	10.871	**0.0001**
Body size	2	23.206	**< 0.0001**
Owner’s dog experience	1	8.213	**0.0042**

All were a priori contrasts. Significant (*P* < 0.05) associations are emboldened. *N* = 9270.

**Figure 1:**
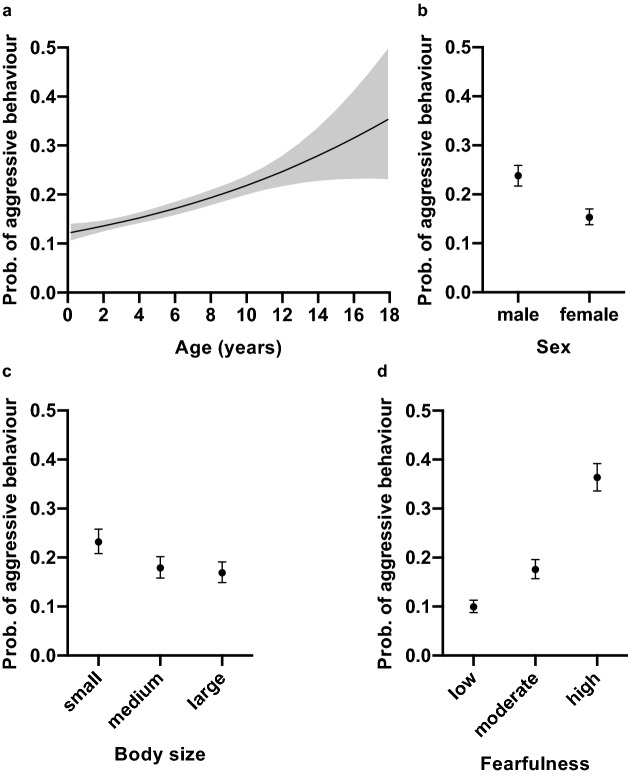
The effect of age, fearfulness, sex, and, body size on aggressive behaviour in the logistic regression analysis. (**a**) Older dogs had a higher probability of aggressive behaviour than young dogs. (**b**) Male dogs had a higher probability of aggressive behaviour than female dogs. (**c**) Small dogs had a higher probability of aggressive behaviour than medium-sized and large dogs. (**d**) Highly and moderately fearful dogs had a higher probability of aggressive behaviour than non-fearful dogs. Grey area (**a**) and error bars (**b**–**d**) indicate 95% confidence limits. *N* = 9270.

The probability of aggressive behaviour correlated positively with age, with older dogs having a higher odds of aggressive behaviour than young dogs (Fig. 1a, Table 1). As hypothesised, male dogs had a higher odds of aggressive behaviour than female dogs (Fig. 1b, Table 2). The dog’s body size was also associated with aggressive behaviour; small dogs had a higher odds of aggressive behaviour than medium-sized and large dogs, but there was no difference between medium-sized and large dogs (Fig. 1c, Table 2). Highly fearful dogs had over five times higher odds of aggressive behaviour than non-fearful dogs and moderately fearful dogs also had a higher odds of aggressive behaviour than non-fearful dogs (Fig. 1d, Table 2).

**Table 2:** Contrasts between different groups of categorical and ordinal variables in the logistic regression analysis.

Contrast	OR	Lower 95% Cl	Upper 95% Cl	*P*-value
**Sex**				
Male vs. female	1.72	1.54	1.93	**< 0.0001**
**Dogs in the family**				
Only dog vs. other dogs	1.23	1.09	1.39	**0.0010**
**Owners dog experience**				
First dog vs. not a first dog	1.21	1.06	1.37	**0.0042**
**Body size**				
Small vs. large	1.488	1.256	1.764	< 0.0001*
Small vs. medium	1.383	1.155	1.658	**0.0041**
Medium vs. large	1.075	0.902	1.282	0.5671
**Fearfulness**				
High vs. low	5.181	4.525	5.917	< 0.0001*
Moderate vs. low	1.931	1.667	2.237	**0.0011**
High vs. medium	2.681	2.342	3.067	**0.0011**
**Breed groups**				
Lagotto Romagnolo, Chihuahua, German Shepherd Dog, and Miniature Schnauzer vs Golden Retriever, and Labrador Retriever	3.185	2.053	4.950	< 0.0001*

*P*-values are controlled for false discovery rate except for a priori contrasts, which were formed after the data collection, but before the analysis. A priori contrasts are marked with *.

Significant *P*-values are bolded (*P*-value < 0.05)

*OR* odds ratio, *Cl* confidence limit, *N* = 9270.

**Figure 2:**
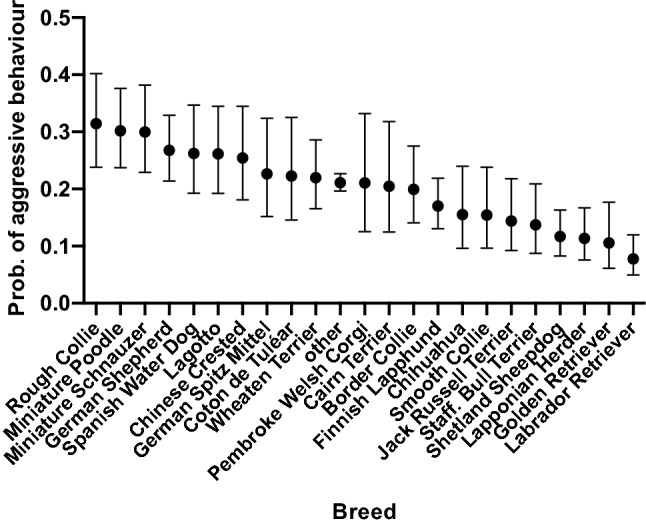
Probability of aggressive behaviour in 23 dog breeds or breed groups. Several breeds differed significantly from each other (Supplementary Table S2). Error bars indicate 95% confidence limits. *N* = 9270.

The probability of aggressive behaviour differed between breeds (Fig. 2). When adjusting for other variables in the model, the breeds with the highest odds of aggressive behaviour were Rough Collie, Miniature Poodle (toy, miniature and medium-sized), and Miniature Schnauzer. The breeds with the lowest odds of aggressive behaviour were Labrador Retriever, Golden Retriever, and Lapponian Herder. As we hypothesised a priori, Lagotto Romagnolo, Chihuahua, German Shepherd Dog, and Miniature Schnauzer had a significantly higher odds for aggressive behaviour than Golden Retriever and Labrador Retriever (Table 2). The largest pairwise differences were found between Rough Collie and Labrador Retriever (OR = 5.44, *P* = 0.0011), Miniature Poodle and Labrador Retriever (OR = 5.13, *P* = 0.0011), and Miniature Schnauzer and Labrador Retriever (OR = 5.08, *P* = 0.0011). Rest of the significant pairwise comparisons between breeds can be seen in the Supplementary Table S2, and all pairwise breed differences are presented in the “Supplementary Dataset”.

In addition to demographic factors, environmental factors also influenced aggressive behaviour. Dogs living without other dogs in the household had a higher odds for aggressive behaviour than dogs living with other dogs (Table 2, Supplementary Fig. S1). In addition, dogs of first-time dog owners had a higher odds of aggressive behaviour (Table 2, Supplementary Fig. S1) than dogs belonging to owners who have had at least one dog previously.

## Discussion

This large-scale survey study of over 9000 pet dogs suggests that aggressive behaviour toward people is affected by behaviour, demography, and environment. The studied factors daily time spent alone, and weaning age were novel, and factors living environment, family size, dogs in the family, dog experience, daily exercise, have previously been studied only in few articles^14,15,20–22^. Dogs showing aggressive behaviour were more often fearful, small-sized, males, owner’s first dogs and the only dogs in the family. In addition, probability of aggressive behaviour increased with age, and we found that the probability of aggressive behaviour differed between dog breeds. These findings suggest that improvements in the owner education and breeding practices of pet dogs could alleviate aggressive behaviour toward people. The identified factors should also be considered when planning studies that aim for the discovery of the associated hereditary factors.

Fearfulness had the strongest association with aggressive behaviour. Fearful and noise-sensitive dogs have been found to behave more aggressively toward unfamiliar people than dogs with no anxieties^11^. In the study of Dinwoodie et al.^28^, the dogs with fear/anxiety problem had more biting incidences than other dogs, and they also found remarkable comorbidity between fear/anxiety and overall aggressive behaviour. Similarly, in the study of Salonen et al.^12^, comorbidity between fearfulness and aggressive behaviour was strong: aggressive dogs were over three times more often fearful than non-aggressive dogs. Aggressive behaviour commonly stems from fearfulness, as fear-related aggressive behaviour is a type of undesired aggressive behaviour^13,29^. Here, we could not separate fear-related aggressive behaviour from other types of aggressive behaviour. Therefore, it is possible that majority of the dogs in this study show fear-related aggressive behaviour.

We found a significant association between sex and aggressive behaviour. Male dogs had a higher probability of aggressive behaviour than females. This association has been found before in some studies^1,28,29^, but Hsu et al.^14^ found this association only with aggressive behaviour toward the owner and Bennett and Rolf^15^ did not find association with unfriendliness/aggressiveness. In addition, in the study population of Guy et al.^30^, female dogs were more likely to have bitten than male dogs. Thus, more studies are needed to reveal the association of sex and aggressive behaviour.

The probability of aggressive behaviour increased with age, and thus, older dogs were more likely aggressive than young dogs. A similar association between age and aggressiveness/unfriendliness has been found earlier^10,15^. However, in the study of Hsu and Sun^14^, age influenced only aggressive behaviour toward the owner, and the difference was significant only when comparing dogs over 10 years of age to dogs under 5 years of age. In contrast, in the study Casey et al.^10^, only the probability of aggressive behaviour toward strangers increased. Study of Col et al.^1^ found no association between age and aggressive behaviour, and it is possible that old dogs have had more opportunities to show aggressive behaviour, reflecting to our finding. As aggressive behaviour can be a sign of pain^5^, it is possible that older dogs have painful conditions or disorders which make them more aggressive. For example, hip dysplasia is a common disease which can cause pain-related aggressive behaviour in dogs^7^. In addition, some disorders, such as the blinding eye disease cataract which is common in older dogs^31^, can decrease the ability to perceive approaching people. This can make the dog feel insecure and increase the chance of an aggressive response. Thus, yearly health checks might reduce pain- or other disease-related aggressive behaviour.

We found differences between dog breeds in the probabilities of aggressive behaviour toward people. From all the studied breeds, Rough Collie had the highest probability of aggressive behaviour. Rough Collies also commonly suffer from another behavioural problem, fearfulness^32^ and thus, it seems that Rough Collies would likely benefit from more behaviour-focused breeding. Besides Rough Collies, other breeds with high probability of aggressive behaviour included the Miniature Poodle, Miniature Schnauzer, German Shepherd Dog, Spanish Water Dog, and Lagotto Romagnolo. In previous studies (Miniature) Poodle^19^ and Miniature Schnauzer^14^ have scored above the average in aggressive behaviour toward strangers, and Lagotto Romagnolo in aggressive behaviour toward family members^11^. The two breeds having the lowest probabilities of aggressive behaviour in our study were Labrador and Golden Retrievers. These breeds have also scored low in previous studies^14,19^. However, some of our breed-wise results differ from previous studies. For example, in the study of Duffy et al.^19^, Chihuahua and Jack Russell Terrier exhibited the most severe signs of aggressive behaviour, such as biting, but in our study, when taking the other factors account (e.g. body size), these breeds were among the least aggressive breeds. Duffy et al.^19^ did not take other factors into account which probably explains the difference between these results. To be noted, Staffordshire Bull Terrier, which is one of the restricted breeds, for example, in Ireland^2^, was not among the most aggressive breeds in this study. In the future, we will also consider breeding lines among the breeds, for example, separate German Shepherd Dog to working and show line types, since the purpose that dogs were bred for can also affect behaviour^33^. Furthermore, some breeds are more prone to, for example, skeletal disorders, which can cause pain-related aggression^34^ and influence these observed breed differences.

Small dogs were more prone to aggressive behaviour than large or medium-sized dogs. Association of small size and aggressive behaviour is in line with some previous studies: taller and heavier dogs were found to be less aggressive toward the owner and strangers than small dogs^17^, and Ley et al.^35^ reported that heavier dogs have higher amicability than lighter dogs. In contrast, Khoshnegah et al.^9^ found that large breeds displayed more aggressive behaviour toward strangers, and Bennett and Rohlf^15^ did not find any association between the dog’s body size and unfriendliness/aggressiveness. To be noted, however, both in our study and in the study of McGreevy et al.^17^, the body size estimates were based on the breed standards, not the actual height of the individuals, which can affect the results. Even though we found no multicollinearity between the breed and body size, we also ran the model without body size and obtained the same results. Thus, we think that the association of body size with aggression mainly comes from the “other breeds” group, which included 6360 individuals from breeds with different body sizes.

Nevertheless, previous studies have also associated small size with fearfulness^9,17,36^ and thus, it seems that small dogs are more vulnerable to behavioural problems in general. Interestingly, owners handle small dogs differently than larger dogs, which can partly explain the higher proportion of behaviour problems in smaller dogs. Owners of small dogs play with and obedience train their dogs less frequently than owners of large dogs^37,38^, and small dogs are also less often house-trained^39^. We speculate that small size can make a dog easier to control even when they act aggressively, and people do not necessary feel threatened by small dogs. Therefore, the owners may not try to treat nor seek professional help for aggressive behaviour so willingly than owners of larger dogs. Professional help, however, have shown to decrease incidence of undesirable behaviours, such as aggression towards strangers, in young dogs^40^. In addition, we speculate that, as people may not feel threatened by small dogs, they might not consider behaviour important when making breeding decisions. Further, a recently published study associated several problematic behaviours with genetic variants known to cause small body size^41^.

The dogs whose owners have had at least one dog before had a lower probability of aggressive behaviour than owners’ first dogs. This finding replicates previously found associations of owner’s dog experience and dominance-type aggressive behaviour^21^ as well as general aggressive behaviour^20^. It is possible that experienced owners are more aware of the importance of socialisation. Previous experience can also help owners to identify a problem at early stage, when the problem can be treated more efficiently. Furthermore, if the owners had problems with their first dogs, they may be more careful when choosing a new dog.

Company of other dogs was associated with a lower probability of aggressive behaviour; dogs living with other dogs were less likely aggressive than dogs living without other dogs. Number of household dogs also decreased aggressive behaviour toward the owner in a study of Hsu and Sun^14^. They suggested that dogs in multi-dog families compete with each other for owners’ attention, with the best behaving dog acquiring more attention and thus, dogs are striving to be obedient. Similarly, dogs living in multi-dog households showed less aggressive behaviour toward the owner and other dogs in a more recent study of Serpell and Duffy^20^. Canine companions may offer something that owners cannot, such as the daily opportunity of intraspecific communication. For example, playing with other dogs could decrease aggressive behaviour emerging from frustration. On the other hand, owners of aggressive dogs may choose not to acquire another dog to avoid possible conflicts between the dogs and ease the handling of the aggressive dog.

This study has some limitations. One of the limitations is that we could not examine aggressive behaviour towards family members and strangers separately due to a small number of dogs showing aggressive behaviour in many breeds. This may affect the reliability of the results, as the study of Salonen et al.^12^ showed distinct breed differences in the aggressive behaviour sub-traits. This also made comparisons between this study and previous ones challenging, because in many other studies aggressive behaviour was divided to sub-traits. In addition, as we did not have any health information from the dogs, we could not identify the individuals having health problems. Owners’ participation to the study was voluntary and thus, the data can be somewhat biased; owners of highly aggressive dog may have not wanted to participate to the study, or, on the other hand, they may have wanted to participate more willingly than owners of non-aggressively behaving dogs. It is also possible that owners did not report all information precisely, for example the breed of the dog. Moreover, as the questionnaire was available only online, participation required basic computer skills and access to the Internet. Finally, this study is cross-sectional and therefore, the causality of the associations discovered cannot be inferred. In the future, it is important to collect even larger datasets, to include health information and to design longitudinal studies, enabling the study of aggressive behaviour sub-traits, associations with health issues and the causal effects.

Our results replicate findings of previous studies in an independent study population and suggest that aggressive behaviour is a complex trait associated with several demographic, environmental, and behavioural factors. The prevalence of aggressive behaviour could be decreased by preferring less aggressive individuals in breeding, since aggressive behaviour has been suggested to be heritable^42,43^. Furthermore, prevalence of aggressive behaviour could also be decreased by using only non-fearful dogs in breeding, as these traits were highly associated and may share a genetic component. Dog owners may decrease the chances of aggressive behaviour by carefully selecting the right breed for their lifestyle and by having multiple dogs. Since aggressive behaviour can be a consequence of pain, yearly health checks could also decrease aggressive behaviour especially in older dogs.

## Methods

### Questionnaire

We used an owner-answered online questionnaire to study aggressive behaviour and collected a cross-sectional convenience sample of Finnish pet dogs. Our survey targeted seven unwanted behaviours in dogs, including fear, aggression, noise sensitivity, fear of surfaces and heights, inattention and hyperactivity/impulsivity, separation anxiety, and compulsive behaviour. The questionnaire also included a comprehensive background section, consisting of questions dealing with the early and current life of the dog and basic demographic information. We advertised the questionnaire to Finnish dog owners in social media, on our website and with the help of breed clubs. We collected the data during 2015–2018. For this study, we used the data from aggressive behaviour, fear, and background sections of the questionnaire. The questionnaire is available as “Supplementary material” in the paper of Salonen et al.^12^ (https://static-content.springer.com/esm/art%3A10.1038%2Fs41598-020-59837-z/MediaObjects/41598_2020_59837_MOESM1_ESM.pdf).

The aggressive behaviour section included two sub-traits, aggressive behaviour toward strangers and aggressive behaviour toward family members. We asked how often the dog growls when a stranger tries to touch or pet it in its home or outside, and how often the dog tries to snap or bite when a stranger tries to touch or pet it in its home or outside. We also asked how often the dog growls when a family member handles the dog or tries to take away a resource (e.g. bone, food or toy) from it, and how often the dog tries to snap or bite when a family member handles the dog or tries to take away a resource from it. The answer was given using a Likert-type scale: 1 = never, 2 = rarely, 3 = sometimes, 4 = often, 5 = always or almost always. Based on the questionnaire answers, we categorised the dogs to low (non-event) and high (event) groups in both sub-traits (aggressive behaviour toward stranger and aggressive behaviour toward family members). We concluded that as biting/snapping is more serious than growling it should have more weight and formed groups based on that. If the dog had tried to bite or snap at least sometimes or growled at least often, it was categorised to the high group. Dogs that bit rarely or growled sometimes were categorised to the moderate group. Dogs that had never shown any of these signs of aggressive behaviour were categorised to the low group. Finally, the dogs were categorised to their final aggressive behaviour group based on their groups in the sub-traits. Dogs that were in the high group in either one of the sub-traits were categorised to the high group. Dogs were categorised to the low group only if they were in the low group in both sub-traits. Dogs that were categorised to the moderate group were excluded, as we used logistic regression in the analysis.

The fear section included three sub-traits, fear of strangers, fear of dogs, and fear of novel situations. We asked how often the dog shows fear in these situations, ranging from never to always using a 5-point Likert-type scale. The sub-sections fear of strangers and fear of novel situations have previously been validated with behavioural tests^25^.

### Statistical analyses

We used logistic regression to examine demographic and environmental factors associated with aggressive behaviour and thus, aggressive behaviour was treated as a binary response variable (event/non-event). For the analyses, we combined sub-traits due to a small number of aggressive dogs in many breeds.

We included several explanatory variables in the analyses, mostly based on previous literature. Explanatory variables included age, sex, sterilisation, breed, body size, weaning age, urban environment score, family size, owner’s dog experience, dogs in the family, daily exercise, daily time spent alone, and fearfulness (Table 3). To study the effect of fearfulness, we divided the dogs into three fearfulness groups (high, moderate, and low) based on the questionnaire. Dogs were categorised to the high group if they had shown fear of strangers, strange dogs or novel situations at least often (40–60% of the times). The moderate group included dogs which have shown fear rarely or sometimes (0–40% of the times), and dogs which have growled or barked when meeting strangers or strange dogs. Dogs were categorised to the low group if the owner had answered that the dog has never shown fear of strangers, strange dogs, or novel situations.

**Table 3:** The variables and their categories used in the model selection of aggressive behaviour.

Variable	Explanation
Aggressive behaviour	Binary (event/non-event) variable. Dogs in the high aggressive behaviour group had tried to bite or snap at least sometimes or growled at least often (event). Dogs in the low aggressive behaviour group had never shown these signs of aggressive behaviour (non-event)
Age	Numerical variable. Reported current age of the dog in years
Sex	Binary variable. Reported sex of the dog. 1: male, 2: female
Sterilisation	Binary variable. Reported status of the dog. 1: intact, 2: neutered
Fearfulness	Ordinal variable. Dogs were divided into three fearfulness groups. High group included dogs which had shown fear of strangers, dogs or novel situations at least often (40–60% of the times). Moderate group included dogs that had shown fear rarely or sometimes (0–40% of the times) or had growled or barked in these situations. Low group included dogs which had never shown fear in these situations
Urban environment score	Numerical variable. The environmental land-use around the dog’s home. The coverage of three land-use types (artificial surfaces, agricultural areas, forests and semi-natural areas) was calculated within a three-kilometre range around the homes. The coverages were simplified into one numerical variable, in which a higher value indicates a more urban environment
Body size	Ordinal variable. Dogs were divided into categories based on the average height of the breed. 1: small (≤ 35 cm), 2: medium (36–49 cm), 3: large (≥ 50 cm)
Breed	Categorical variable. Reported breed of the dog. Border Collie, Cairn Terrier, Chihuahua (short haired and long haired), Chinese Crested Dog, Coton de Tulèar, Finnish Lapponian Dog, German Shepherd Dog, Golden Retriever, Irish Soft Coated Wheaten Terrier (labelled Wheaten Terrier), Jack Russell Terrier, Labrador Retriever, Lagotto Romagnolo, Lapponian Herder, Medium size Spitz, Miniature Poodle (toy, miniature, and medium sized), Miniature Schnauzer, Pembroke Welsh Corgi, Rough Collie, Shetland Sheepdog, Smooth Collie, Spanish Water Dog, Staffordshire Bull Terrier, other
Weaning age	Ordinal variable. The reported weaning ages were divided into four categories. 1: < 7 weeks of age, 2: at 7 weeks of age, 3: at 8 weeks of age, 4: > 8 weeks of age
Family size	Ordinal variable. The size of the family in which the dog lives. 1: single, 2: couple, 3: family with one or two adults and one child, 4: family with one or two adults and two children, 5: family with three or more adults and/or three or more children
Dogs in the family	Binary variable. Describes whether there are other dogs in the family. 1: the dog is the only dog in the family, 2: the dog lives with one or more dogs
Owner’s dog experience	Binary variable. Describes owner’s experience with dogs. 1: the dog is the owner’s first dog, 2: the owner has had dogs before this dog
Daily exercise	Ordinal variable. Describes the amount of dog’s daily exercise in hours. 1: < 1 h, 2: 1–2 h, 3: 2–3 h, 4: > 3 h
Daily time spent alone	Ordinal variable. Describes the daily time that dog spends alone at home without the presence of people. 1: < 3 h, 2: 3–6 h, 3: 6–8 h, 4: > 8 h

To study the effect of dog’s body size on aggressive behaviour, we formed size groups using FCI and AKC breed standards, when available. If female and male dogs had a different height standard within the breed, we calculated the mean height. According to the heights, we divided the dog breeds into three size groups: small (≤ 35 cm), medium (36–49 cm), and large (≥ 50 cm). As heights could not be determined for mixed breed dogs (N = 114), we excluded them from the analysis. We selected 22 breeds with adequate sample sizes for the analysis (Table 3) in addition to “other” breed group which included individuals from breeds with less than ten individuals per aggressive behaviour group. Based on the weaning age (age when the dog was separated from its mother), we divided the dogs into four groups; early weaned (< 7 weeks), normally weaned (7 weeks and 8 weeks), and late weaned (> 8 weeks) group. We excluded dogs still living with their dam.

We calculated the urban environment score for the dog’s daily living environment based on the geographical coordinates of owner’s home addresses. To do this, we first determined the land-use within a three-kilometre radius around the dog’s home in three land-use types: artificial surfaces, agricultural areas, and forests and semi-natural areas, using the land-use database CORINE2012 with a 25 m resolution. Land use describes the utilisation of land, including the management of natural environment and modification of it into built environment such as settlements. Next, we transformed the land-use information into one continuous variable with principal component analysis (PCA). This simplified the land-use to a rural–urban gradient (labelled urban environment score), with higher values indicating a more urban environment. For example, the dog who had the highest urbanization score lived in the city centre of the capital of Finland, and the dog who had the lowest score lived in the countryside, surrounded by forests and fields.

Initially, the questionnaire data included 13,715 dogs. Dogs with high (event) or low (non-event) aggressive behaviour and no missing responses in the studied explanatory variables were included, leading to a dataset of 5511 dogs. Our starter model for logistic regression included the dog’s age and sex as explanatory variables. In addition, we included several other explanatory variables (Table 3), mostly based on the previous literature. We chose the model with the best fit using a forward stepwise Akaike Information Criterion (AIC) selection approach. The explanatory variables fearfulness, breed, dogs in the family, body size, and owner’s dog experience improved model fit and were included in the final model. In contrast, the explanatory variables weaning age, sterilisation, daily exercise, time spend alone, family size, and urban environment score did not improve model fit and were discarded. The model selection is shown in the Supplementary Table S3. After the model selection, we maximised the use of data by including all dogs that had missing responses only in the explanatory variables that did not end up to the final model. For example, dogs who had missing responses in weaning age were included in the final model. We compared the ANOVA tables of smaller and larger data sets to ensure the model did not essentially change from the inclusion of additional dogs, and the tables were extremely similar. Thus, the final data consisted of 9270 dogs. R 3.5.2 was used in all analyses^44^.

After the model selection, we inspected the linearity assumption of numerical variables by fitting a generalised additive model with the package ‘gam’^45^. The explanatory variable age did not meet the assumption, and thus we included age as a linear and a quadratic (age^2) variable in the final model. Next, we inspected possible outliners with packages ‘broom’^46^ and ‘dplyr’^47^. We plotted standardised residuals using package ‘ggplot2’^48^, and tested the multicollinearity with package ‘car’^49^ with generalised variance inflation factor (gVIF). There was no multicollinearity, but we identified three outliers. Removing these outliers did not affect the results and as they were actual responses, we kept them in the final data. Finally, we calculated the area under the receiver operator characteristic curve (AUC) using package ‘pROC’^50^ to estimate how well the model predicts the event (high aggressive behaviour group) and non-event (low aggressive behaviour group). The AUC of the final model was 0.74.

Based on previous literature, we had several hypotheses and we formed multiple a priori contrasts between the categories of the explanatory variables. Our approach was exploratory, and we formed hypotheses after the data collection, but before the data analysis. We hypothesised that older dogs are more aggressive than younger dogs^10,14,15^, that male dogs are more aggressive than female dogs^1,14^, and that small sized dogs are more aggressive than larger dogs^17^. We also hypothesised that highly fearful dogs are more aggressive than non-fearful individuals^11,13^, that dogs living in households without other dogs are more aggressive than dogs living with other dogs^20^, that dogs living in rural areas are more aggressive than ones living in cities^14^, that early weaned dogs are more aggressive than late weaned dogs^23^, and that dogs living with unexperienced owners have a higher probability of aggressive behaviour^20–22^. We also hypothesised that Lagotto Romagnolo, Chihuahua, German Shepherd Dog, and Miniature Schnauzer are more aggressive breeds than Golden Retriever and Labrador Retriever^11,14,19,20^.

To calculate the estimated marginal means for categorical and ordinal explanatory variables, we used the package ‘emmeans’^51^. To obtain the means and confidence limits of numerical explanatory variables, we used the package ‘effects’^52^, and to see the overall effect of all explanatory factors, we conducted analysis of variance (ANOVA) with the package ‘car’^49^. For other than the hypothesised contrast chosen a priori, we corrected the obtained *P*-values for false discovery rate (FDR). The significance cut-off was set at *P*-value < 0.05. All methods were carried out in accordance with local guidelines and regulations.

### Ethics statement

The data was collected before the onset of the GDPR regulation according to the Finnish legislation: https://www.finlex.fi/fi/laki/ajantasa/1999/19990523. This survey study focused on dogs and not human participants or the dog owners, and therefore a specific ethical approval was not needed at that time for academic research studies. As for the study participants (dog owners), we collected only names and addresses for contacting the owners in subsequent studies and for calculating the urban-environment score.

Owners were informed that the participation is voluntary, confidential, and that the data is used only for scientific purposes. In addition, an information sheet was provided to all participants. We received informed consent from all participants.

## Supplementary Information

Supplementary Dataset.

Supplementary Information.

## Data Availability

The anonymised data is available as a “Supplementary file” in the paper of Salonen et al.^12^.
