# Supplementary material for: Aggressive behaviour is affected by demographic, environmental and behavioural factors in purebred dogs
Source: Sci Rep. 2021 May 3;11:9433. doi: 10.1038/s41598-021-88793-5 (PMC8093277; doi:10.1038/s41598-021-88793-5)
Supplement: Supplementary file 2 — Supplementary Information. [file 41598_2021_88793_MOESM2_ESM.docx]

Supplementary Information

Aggressive behaviour is affected by demographic, environmental and behavioural factors in purebred dogs

Salla Mikkola^1,2,3^, Milla Salonen, ^1,2,3^ Jenni Puurunen^1,2,3^, Emma Hakanen^1,2,3^, Sini Sulkama^1,2,3^, César Araujo^1,2,3^, Hannes Lohi^1,2,3*^

^1^ Department of Medical and Clinical Genetics, University of Helsinki, Helsinki, Finland

^2^ Department of Veterinary Biosciences, University of Helsinki, Helsinki, Finland

^3^ Folkhälsan Research Center, Helsinki, Finland

* Corresponding author:

Hannes Lohi, PhD, Professor

Email: hannes.lohi@helsinki.fi (HL)

Supplementary Table S1. Number of dogs in different breeds, sexes, and aggressive behaviour groups.

|  | **High aggressive behaviour** | | | **Low aggressive behaviour** | | |  |
| --- | --- | --- | --- | --- | --- | --- | --- |
| **Breed** | Males | Females | Total | Males | Females | Total | **Total** |
| Finnish Lapponian Dog | 38 | 21 | 59 | 156 | 181 | 337 | 396 |
| Labrador Retriever | 11 | 8 | 19 | 141 | 194 | 335 | 354 |
| German Shepherd Dog | 36 | 37 | 73 | 123 | 143 | 266 | 339 |
| Shetland Sheepdog | 21 | 14 | 35 | 108 | 145 | 253 | 288 |
| Wheaten Terrier | 27 | 21 | 48 | 91 | 121 | 212 | 260 |
| Lapponian Herder | 16 | 9 | 25 | 99 | 99 | 198 | 223 |
| Border Collie | 15 | 15 | 30 | 77 | 101 | 178 | 208 |
| Miniature Poodle | 39 | 22 | 61 | 54 | 80 | 134 | 195 |
| Smooth Collie | 26 | 18 | 44 | 60 | 74 | 134 | 178 |
| Lagotto Romagnolo | 22 | 18 | 40 | 47 | 76 | 123 | 163 |
| Miniature Schnauzer | 36 | 22 | 58 | 47 | 51 | 98 | 156 |
| Staffordshire Bull Terrier | 10 | 9 | 19 | 67 | 70 | 137 | 156 |
| Spanish Water Dog | 22 | 19 | 41 | 45 | 59 | 104 | 145 |
| Golden Retriever | 4 | 9 | 13 | 52 | 78 | 130 | 143 |
| Rough Collie | 8 | 9 | 17 | 62 | 60 | 122 | 139 |
| Jack Russell Terrier | 14 | 8 | 22 | 49 | 41 | 90 | 112 |
| Chinese Crested Dog | 23 | 17 | 40 | 34 | 32 | 66 | 106 |
| Medium size Spitz | 17 | 9 | 26 | 40 | 27 | 67 | 93 |
| Coton de Tulèar | 10 | 14 | 24 | 27 | 34 | 61 | 85 |
| Chihuahua | 14 | 6 | 20 | 32 | 29 | 61 | 81 |
| Cairn Terrier | 7 | 10 | 17 | 26 | 26 | 52 | 69 |
| Pembroke Welsh Corgi | 7 | 7 | 14 | 23 | 30 | 53 | 67 |
| Other | 594 | 452 | 1046 | 1903 | 2365 | 4268 | 5314 |

Supplementary Table S2. Significant breed differences in the logistic regression analysis. *N* = 9,270. All *P*-values are FDR-controlled. OR = odds ratio, Cl = confidence limit.

| **Breed contrast** | **OR** | **Lower 95% Cl** | **Upper 95% Cl** | ***P*-value** |
| --- | --- | --- | --- | --- |
| Border Collie vs. Labrador Retriever | 2.957 | 1.590 | 5.499 | 0.0046 |
| Cairn Terrier vs. Labrador Retriever | 3.058 | 1.421 | 6.581 | 0.0210 |
| Chihuahua vs. Miniature Poodle | 0.424 | 0.226 | 0.794 | 0.0310 |
| Chihuahua vs. Miniature Schnauzer | 0.428 | 0.227 | 0.808 | 0.0360 |
| Chihuahua vs. Rough Collie | 0.400 | 0.206 | 0.780 | 0.0308 |
| Chinese Crested Dog vs. Golden Retriever | 2.892 | 1.380 | 6.063 | 0.0228 |
| Chinese Crested Dog vs. Labrador Retriever | 4.048 | 2.110 | 7.769 | 0.0011 |
| Chinese Crested Dog vs. Lapponian Herder | 2.661 | 1.423 | 4.974 | 0.0127 |
| Chinese Crested Dog vs. Shetland Sheepdog | 2.575 | 1.437 | 4.614 | 0.0095 |
| Coton de Tulèar vs. Labrador Retriever | 3.400 | 1.671 | 6.921 | 0.0052 |
| Finnish Lapponian Dog vs. Miniature Poodle | 0.473 | 0.304 | 0.737 | 0.0065 |
| German Shepherd Dog vs. Finnish Lapponian Dog | 1.784 | 1.156 | 2.753 | 0.0360 |
| German Shepherd Dog vs. Shetland Sheepdog | 2.759 | 1.691 | 4.501 | 0.0011 |
| German Shepherd Dog vs. Staffordshire Bull Terrier | 2.298 | 1.268 | 4.164 | 0.0269 |
| Golden Retriever vs. German Shepherd Dog | 0.323 | 0.169 | 0.617 | 0.0046 |
| Golden Retriever vs. Lagotto Romagnolo | 0.333 | 0.162 | 0.686 | 0.0151 |
| Golden Retriever vs. Miniature Poodle | 0.273 | 0.137 | 0.542 | 0.0022 |
| Golden Retriever vs. Other | 0.441 | 0.242 | 0.802 | 0.0310 |
| Jack Russell Terrier vs. German Shepherd Dog | 0.460 | 0.256 | 0.827 | 0.0374 |
| Jack Russell Terrier vs. Miniature Poodle | 0.389 | 0.214 | 0.705 | 0.0118 |
| Jack Russell Terrier vs. Miniature Schnauzer | 0.392 | 0.215 | 0.717 | 0.0130 |
| Labrador Retriever vs. Finnish Lapponian Dog | 0.411 | 0.231 | 0.734 | 0.0144 |
| Labrador Retriever vs. German Shepherd Dog | 0.231 | 0.134 | 0.397 | 0.0011 |
| Labrador Retriever vs. Irish Soft Coated Wheaten Terrier | 0.299 | 0.164 | 0.545 | 0.0011 |
| Labrador Retriever vs. Lagotto Romagnolo | 0.238 | 0.127 | 0.447 | 0.0011 |
| Labrador Retriever vs. Medium size Spitz | 0.288 | 0.144 | 0.576 | 0.0036 |
| Labrador Retriever vs. Miniature Poodle | 0.195 | 0.108 | 0.351 | 0.0011 |
| Labrador Retriever vs. Other | 0.315 | 0.194 | 0.511 | 0.0011 |
| Labrador Retriever vs. Pembroke Welsh Corgi | 0.316 | 0.143 | 0.696 | 0.0211 |
| Lagotto Romagnolo vs. Lapponian Herder | 2.759 | 1.553 | 4.899 | 0.0041 |
| Lagotto Romagnolo vs. Shetland Sheepdog | 2.670 | 1.575 | 4.525 | 0.0029 |
| Lagotto Romagnolo vs. Staffordshire Bull Terrier | 2.224 | 1.189 | 4.159 | 0.0463 |
| Lapponian Herder vs. German Shepherd Dog | 0.351 | 0.205 | 0.601 | 0.0011 |
| Lapponian Herder vs. Irish Soft Coated Wheaten Terrier | 0.455 | 0.264 | 0.783 | 0.0217 |
| Lapponian Herder vs. Miniature Poodle | 0.296 | 0.172 | 0.511 | 0.0011 |
| Lapponian Herder vs. Other | 0.479 | 0.305 | 0.753 | 0.0093 |
| Miniature Schnauzer vs. Finnish Lapponian Dog | 2.091 | 1.286 | 3.401 | 0.0153 |
| Miniature Schnauzer vs. Golden Retriever | 3.632 | 1.797 | 7.341 | 0.0029 |
| Miniature Schnauzer vs. Labrador Retriever | 5.084 | 2.766 | 9.344 | 0.0011 |
| Miniature Schnauzer vs. Lapponian Herder | 3.342 | 1.867 | 5.980 | 0.0011 |
| Miniature Schnauzer vs. Other | 1.601 | 1.107 | 2.316 | 0.0464 |
| Miniature Schnauzer vs. Shetland Sheepdog | 3.234 | 1.890 | 5.534 | 0.0011 |
| Miniature Schnauzer vs. Staffordshire Bull Terrier | 2.694 | 1.429 | 5.079 | 0.0127 |
| Other vs. Miniature Poodle | 0.618 | 0.442 | 0.865 | 0.0233 |
| Other vs. Shetland Sheepdog | 2.020 | 1.366 | 2.986 | 0.0036 |
| Rough Collie vs. Finnish Lapponian Dog | 2.236 | 1.360 | 3.677 | 0.0095 |
| Rough Collie vs. Golden Retriever | 3.885 | 1.944 | 7.764 | 0.0011 |
| Rough Collie vs. Jack Russell Terrier | 2.725 | 1.444 | 5.143 | 0.0121 |
| Rough Collie vs. Labrador Retriever | 5.438 | 2.997 | 9.865 | 0.0011 |
| Rough Collie vs. Lapponian Herder | 3.574 | 1.978 | 6.456 | 0.0011 |
| Rough Collie vs. Other | 1.712 | 1.169 | 2.509 | 0.0260 |
| Rough Collie vs. Shetland Sheepdog | 3.459 | 2.002 | 5.975 | 0.0011 |
| Rough Collie vs. Staffordshire Bull Terrier | 2.882 | 1.516 | 5.475 | 0.0082 |
| Shetland Sheepdog vs. Irish Soft Coated Wheaten Terrier | 0.470 | 0.287 | 0.771 | 0.0149 |
| Shetland Sheepdog vs. Miniature Poodle | 0.306 | 0.186 | 0.503 | 0.0011 |
| Smooth Collie vs. Miniature Poodle | 0.422 | 0.224 | 0.795 | 0.0314 |
| Smooth Collie vs. Miniature Schnauzer | 0.427 | 0.222 | 0.818 | 0.0404 |
| Smooth Collie vs. Rough Collie | 0.399 | 0.210 | 0.756 | 0.0228 |
| Spanish Water Dog vs. Golden Retriever | 3.016 | 1.463 | 6.219 | 0.0149 |
| Spanish Water Dog vs. Labrador Retriever | 4.222 | 2.245 | 7.942 | 0.0011 |
| Spanish Water Dog vs. Lapponian Herder | 2.775 | 1.558 | 4.941 | 0.0041 |
| Spanish Water Dog vs. Shetland Sheepdog | 2.686 | 1.580 | 4.564 | 0.0029 |
| Spanish Water Dog vs. Staffordshire Bull Terrier | 2.237 | 1.194 | 4.194 | 0.0459 |
| Staffordshire Bull Terrier vs. Miniature Poodle | 0.367 | 0.202 | 0.670 | 0.0077 |

Supplementary Table S3. AIC model selection in the logistic regression analysis. *N* = 5,511. The bolded values indicate models that had the best fit in each model selection round.

| Model | AIC | Fearfulness  added | Breed  added | Dogs in  the family  added | Body size  added |
| --- | --- | --- | --- | --- | --- |
| Basic model (sex, age) | 5315.0 |  |  |  |  |
| Fearfulness | **4896.9** |  |  |  |  |
| Breed | 5258.9 | **4850.5** |  |  |  |
| Dogs in the family | 5290.9 | 4885.8 | **4838.7** |  |  |
| Body size | 5256.2 | 4870.4 | 4841.0 | **4830.5** |  |
| Owner’s dog experience | 5295.5 | 4887.1 | 4839.9 | 4834.2 | **4827.5** |
| Urban environment score | 5309.2 | 4897.3 | 4851.2 | 4840.7 | 4832.5 |
| Sterilisation | 5297.5 | 4894.5 | 4847.9 | 4836.5 | 4828.1 |
| Weaning age | 5320.7 | 4900.9 | 4851.3 | 4839.0 | 4830.1 |
| Daily exercise | 5295.8 | 4890.9 | 4845.4 | 4834.6 | 4828.2 |
| Family size | 5313.7 | 4897.5 | 4852.1 | 4839.5 | 4831.0 |
| Daily time spent alone | 5316.4 | 4899.6 | 4853.9 | 4842.6 | 4834.5 |
|  |  |  |  |  |  |

Final model: sex, age, fearfulness, breed, dogs in the family, body size, owner’s dog experience


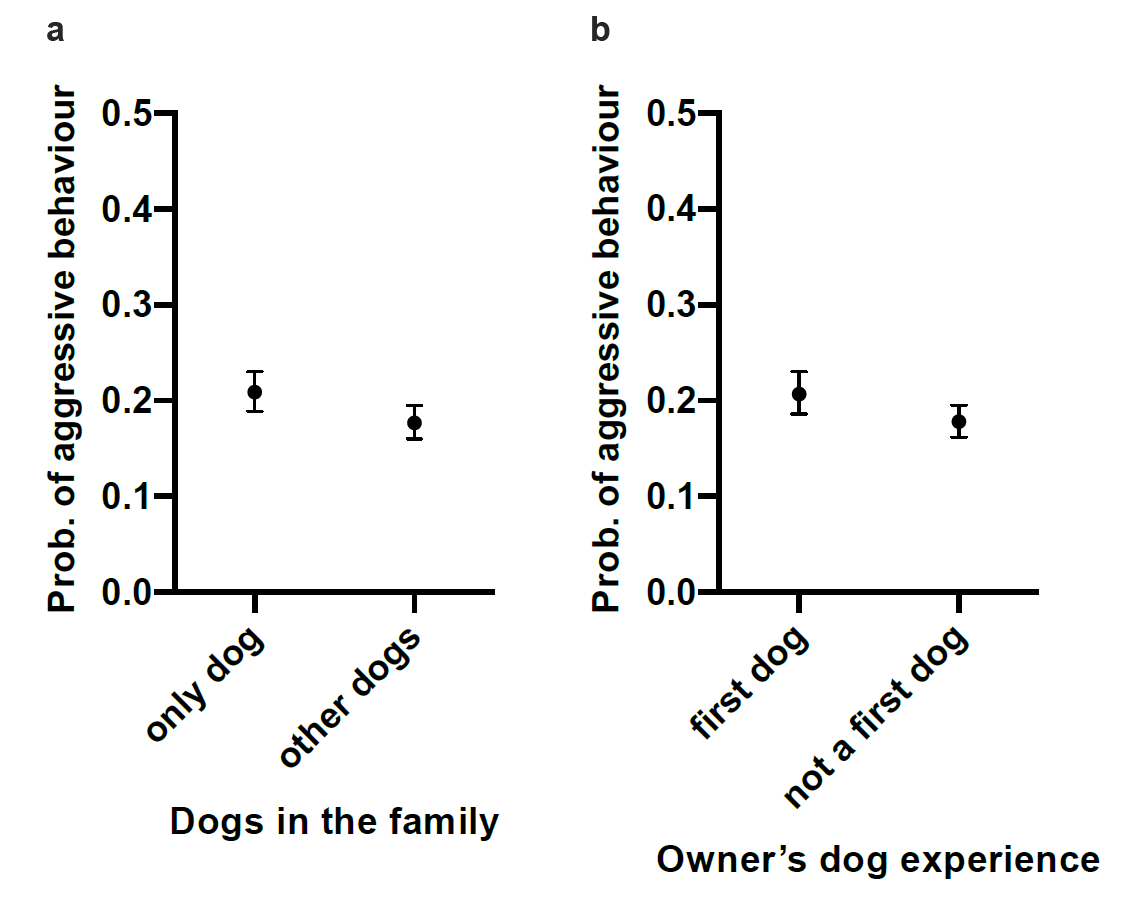


Supplementary Figure S1. The effect of dogs in the family and owner’s dog experience on aggressive behaviour in the logistic regression analysis. (a) Only dogs in the family had increased probability of aggressive behaviour. (b) Owner’s previous dog experience decreased the probability of aggressive behaviour. Error bars indicate 95% confidence limits. *N* = 9,270.
